# Acceptability of a theory-based sedentary behaviour reduction intervention for older adults (‘On Your Feet to Earn Your Seat’)

**DOI:** 10.1186/s12889-015-1921-0

**Published:** 2015-07-02

**Authors:** Raluca Matei, Ingela Thuné-Boyle, Mark Hamer, Steve Iliffe, Kenneth R. Fox, Barbara J. Jefferis, Benjamin Gardner

**Affiliations:** Health Behaviour Research Centre, University College London, London, UK; Population Health Domain Physical Activity Research Group, Department of Epidemiology and Public Health, University College London, London, UK; Research Department of Primary Care & Population Health, University College London, London, UK; Centre for Exercise, Nutrition and Health Sciences, University of Bristol, Bristol, UK; Department of Psychology, Institute of Psychiatry, Psychology and Neuroscience, King’s College London, 9th Floor, Capital House, 42 Weston Street, London, SE1 3QD UK; UCL Centre for Behaviour Change, University College London, London, UK

**Keywords:** Older adults, Sedentary behaviour, Sitting, Physical activity, Intervention, Habit, Behaviour change

## Abstract

**Background:**

Adults aged 60 years and over spend most time sedentary and are the least physically active of all age groups. This early-phase study explored acceptability of a theory-based intervention to reduce sitting time and increase activity in older adults, as part of the intervention development process.

**Methods:**

An 8-week uncontrolled trial was run among two independent samples of UK adults aged 60–75 years. Sample 1, recruited from sheltered housing on the assumption that they were sedentary and insufficiently active, participated between December 2013 and March 2014. Sample 2, recruited through community and faith centres and a newsletter, on the basis of self-reported inactivity (<150 weekly minutes of moderate-to-vigorous activity) and sedentary behaviour (≥6 h mean daily sitting), participated between March and August 2014. Participants received a booklet offering 16 tips for displacing sitting with light-intensity activity and forming activity habits, and self-monitoring ‘tick-sheets’. At baseline, 4-week, and 8-week follow-ups, quantitative measures were taken of physical activity, sedentary behaviour, and habit. At 8 weeks, tick-sheets were collected and a semi-structured interview conducted. Acceptability was assessed for each sample separately, through attrition and adherence to tips, ANOVAs for behaviour and habit changes, and, for both samples combined, thematic analysis of interviews.

**Results:**

In Sample 1, 12 of 16 intervention recipients completed the study (25 % attrition), mean adherence was 40 % (per-tip range: 15–61 %), and there were no clear patterns of changes in sedentary or physical activity behaviour or habit. In Sample 2, 23 of 27 intervention recipients completed (15 % attrition), and mean adherence was 58 % (per-tip range: 39–82 %). Sample 2 decreased mean sitting time and sitting habit, and increased walking, moderate activity, and activity habit. Qualitative data indicated that both samples viewed the intervention positively, found the tips easy to follow, and reported health and wellbeing gains.

**Conclusions:**

Low attrition, moderate adherence, and favourability in both samples, and positive changes in Sample 2, indicate the intervention was acceptable. Higher attrition, lower adherence, and no apparent behavioural impact among Sample 1 could perhaps be attributable to seasonal influences. The intervention has been refined to address emergent acceptability problems. An exploratory controlled trial is underway.

**Electronic supplementary material:**

The online version of this article (doi:10.1186/s12889-015-1921-0) contains supplementary material, which is available to authorized users.

## Background

The importance of regular physical activity (PA) for health has been recognised for many years [1]. A more recent, growing literature suggests sitting time may be a risk factor for physical and mental health, independently of engagement in physical activity [2–6]. Researchers and practitioners have called for a distinction between sedentary behaviour (SB; i.e., low-energy expending behaviour undertaken while seated or lying down [7]) and physical inactivity (i.e. insufficient PA). Public health guidelines increasingly recommend not only regular moderate-to-vigorous PA (MVPA), but also minimization of sitting time, and interruption of prolonged sitting with regular standing or activity breaks [8–10].

Both regular PA, and minimising SB, are thought to confer independent cardio-metabolic health benefits throughout the life span [11, 12]. There may be additional benefits for older adults, in that increasing PA and reducing SB may shield against declines in physical and mental health and wellbeing [13]. Although studies have yielded mixed results and generally been of low-quality [14], there is nonetheless some evidence of links between SB and dementia [15], and frailty-related falls [16]. Yet, of all age groups, older adults on average spend most time sedentary, and least time physically active [17]. One national survey suggested that, of people aged 65–74 in England, 55 % spent six or more daily hours in SB, and 30 % had not performed any MVPA for at least 10 consecutive minutes over the preceding four weeks [17]. Behaviour change interventions are needed to reduce SB in older adults. Four forms of PA are recommended for older adults: aerobic, balance, flexibility, and muscle-strengthening [9, 18]. Standing is a form of light balance activity that may have health benefits [19], and so reducing SB necessarily entails increases in at-least-light PA. Effective SB reduction interventions in older adults might therefore confer health benefits arising not only from reduced sitting time, but also from increasing PA.

While declines in physical functioning in the ‘oldest old’ (roughly, those aged over 75 years) may impose limits on PA, replacing SB with PA of at least light intensity is likely to be feasible among the ‘young-old’ (roughly, 60–75 years) [20]. The few interventions that have explicitly targeted SB reduction among older adults have yielded promising results [21–23]. Uncontrolled pilot evaluations of two interventions involving provision of individualized consultations and personalized accelerometer feedback to older adults showed reductions in sitting time and increases in light PA and MVPA [21, 22]. A smartphone application designed to educate older adults of the risks of SB, and provide real-time SB and PA feedback, was found to significantly decrease daily TV viewing [23], one of the most prevalent sedentary activities among older adults [24]. These studies demonstrate the feasibility of displacing sedentary activities with PA among older adults through purposive intervention.

We have developed a theory- and evidence-based intervention that seeks to displace SB with PA among older adults [25]. Designed for, and in partnership with, adults aged 60–75 years (the ‘young old’), the intervention centres on a booklet (titled ‘On Your Feet to Earn Your Seat’) outlining the importance of minimizing SB and taking regular PA in older adulthood, and offering recommendations (‘tips’) for specific actions that may be undertaken to achieve SB reduction. The intervention is based on habit theory, and aims to promote formation of habits for at-least-light PA in place of existing SB habits. It thus conceptually replicates the ‘Ten Top Tips’ intervention, a habit-based leaflet outlining dietary and physical activity tips that has shown potential for promoting weight loss [26, 27]. The present intervention focuses on at-least-light PA because the greatest public health gains from PA promotion may arise from shifting the large proportion of highly inactive individuals into doing some PA, even if the resultant PA falls short of the recommended 150 min MVPA per week [28]. Actions were selected for inclusion on the basis that they could be easily incorporated into existing sedentary routines, so as to become automatically triggered by everyday events (i.e. habitual) [29–32].

Our intervention has been developed in accordance with UK Medical Research Council complex intervention guidelines [33]. These argue for the importance of undertaking pilot work to investigate the feasibility of implementing and assessing an intervention as intended. Feasibility is in part determined by acceptability: interventions deemed unacceptable by the target population – that is, intended recipients would not be willing to receive or adhere to them – are unlikely to be implementable [34]. Small-scale studies of intervention acceptability can play a valuable role in the intervention development process, by pointing to areas that may require refinement ahead of a costly controlled trial [33].

### The present study

This paper describes a mixed-methods analysis of the acceptability of a SB-reduction and PA-promotion intervention for older adults, drawn from an 8-week uncontrolled study. The intervention was designed to displace SB with light-intensity PA and so, if effective, sedentary and highly inactive older adults would likely derive most benefit. Nonetheless, any sedentary older adult could potentially benefit from SB reduction, regardless of activity levels. Thus, we sought to explore acceptability among two independent samples of sedentary adults, intended to represent differing levels of inactivity.

This study was conducted as part of the intervention development process, and was a precursor to a controlled trial, the protocol for which has been published elsewhere [25]. The aim of the present study was to explore intervention acceptability, which was addressed by documenting (a) attrition rates, (b) adherence to each tip, (c) changes in SB and PA behaviour and habit strength, and (d) participants’ views towards the intervention.

## Methods

### Intervention

#### Theory basis

The present intervention draws on the habit-formation model [30, 32]. Habitual behaviours are controlled by impulses that are generated automatically when encountering situations in which the behaviour has been performed in the past [29]. Habitual behaviours are thought to self-perpetuate, and can prompt behaviour even where conscious motivation to do the behaviour is low [35]. Habit-formation has thus been proposed as a potential mechanism for sustaining new behaviours over time, by shielding them against common long-term losses in motivation that threaten to reverse short-term behaviour gains [36]. Habit forms when a given behaviour is repeated in a particular context, because this strengthens the mental association between context and behaviour [23]. Habit-formation advice is based on repeating a chosen behaviour in a setting until it becomes automatic [30]. It differs from non-habit-based advice in specifying not only *which* behaviour should be adopted, but also *how* it might be performed so as to aid maintenance [23]. Forming a habit requires the motivation and volitional skills and resources to sustain behavioural repetition until the behaviour becomes automatic [32]. Repetition is best facilitated by pursuing behaviours that are manageable and realistic [37], and by self-monitoring behaviour [32]. Habit is thought to form more quickly for simple actions [31]. Habit-formation interventions may therefore be most effective where paired with a ‘small changes’ approach to behaviour change, based on making minor modifications to existing practices rather than major changes [30].

Lally and Gardner’s [32] habit-formation framework was used to guide the selection of behaviour change techniques for the intervention. Specifically, techniques were chosen to: enhance motivation to reduce SB and increase PA; facilitate the translation of motivation into action; and promote and sustain repeated performance of PA, or disruption of SB, in consistent contexts. The intervention was co-designed by a panel of 15 experts (covering sports and exercise science, ageing, geriatrics, general practice, psychology, physiology, and physiotherapy), and two independent panels (Ns = 17 and 23) of self-reportedly inactive (< 30 mins leisure time MVPA per week) and sedentary (> 6 leisure time hours spent sitting per day) retired adults aged 60–75 years. Further intervention development detail has been provided elsewhere [25].

#### Intervention content

The present intervention comprised an information booklet, outlining the health risks of SB and benefits of PA, and offering tips and rationale for undertaking PA in a way that would reduce SB or build PA habits, as supplemented by a set of tick-sheets to record adherence to each tip (for both intervention and data collection purposes). The tips were designed to promote all four recommended forms of PA in older adulthood (aerobic, balance, flexibility, muscle-strengthening) and reduce SB. Where possible, tips specified an everyday contextual cue (e.g. ‘during breaks between TV programmes…’) and recommended a behaviour for enactment in the presence of the cue (‘…stand up and walk around’), with justification relating to health or wellbeing (‘this will stop your joints from seizing up’). This format was used to promote motivation to perform the action and the context-dependent repetition necessary for habit to form [32]. ‘Handy hints’ were offered with most tips to provide instructions, offer less or more intensive variants of the recommended activity, or suggest preparatory or supplementary actions likely to increase likelihood of enactment (e.g. ‘leave the remote control by the TV so that you have to get up to change channel’). Text at the end of the booklet described behaviour change techniques conducive to habit formation (e.g. ‘plan ahead’ [action planning], ‘track your progress’ [self-monitoring], ‘start low, go slow’ [graded tasks]) [32]. The booklet recommended adhering to as many tips as possible, while not attempting anything that felt uncomfortable or that a physician had advised against. An extensive description of intervention content, coded according to component behaviour change techniques [38], is provided in Additional file 1: Table S1.

The intervention was presented in an information-only leaflet-based format because, while such interventions are often assumed to be ineffective [39], a leaflet providing habit-formation advice has previously shown efficacy for changing behaviour [26, 27]. This suggests that information content, not delivery method, may determine effectiveness. Previous studies have shown written advice on context-consistent repetition of simple actions to be novel, motivating and acceptable to participants [27, 40].

#### Intervention cost

Excluding an initial charge of £1050 (~US $1600) for visual design and typesetting, each intervention booklet as supplemented by 8 ticksheets cost £4.50 (~US $7), which covered printing costs only. No other intervention costs were incurred.

### Study design

An uncontrolled (pre-post) intervention design was used, with two independent samples, with three data collection time points over an 8-week study period (baseline, 4-weeks, 8-weeks). All study procedures were undertaken with Sample 1 by a registered post-doctoral health psychologist, and with Sample 2 by a fully-trained MSc Health Psychology student. Ethical approval was provided by the University College London Research Ethics Committee.

### Procedure

#### Eligibility criteria

In both samples, participants were eligible only where they reported being aged 60–75 years^1^, able to speak and understand English, and with no physical impairments precluding engagement in light PA. No explicit PA or SB criteria were imposed on Sample 1. In Sample 2, participants were only eligible where they self-reported ≥ 6 h of mean daily sitting time, and < 150 total weekly minutes of MVPA, over a typical week. These criteria were used to ensure that all participants in Sample 2 were sedentary and insufficiently active according to national guidelines, characteristics assumed of Sample 1 based on previous literature [41]. Participants who self-reported any mental health problem were excluded, because the researchers were not qualified to assess capacity to provide informed consent. Eligibility was based on participant self-report only; no further screening (e.g. physical health) took place.

#### Recruitment

Sample 1 was recruited from sheltered housing sites, which are self-contained flats within a larger building, with communal areas for socialization, and warden assistance, for adults aged 55 or over who are less able to live independently. This group was purposefully selected because sheltered housing residents tend to have higher rates of physical inactivity than those living independently [41], and so were targeted to reflect the least active subgroup of older adults, who would derive most benefit from intervention. Sample 2 was recruited from community settings, purposefully selected on the basis of SB, and physical inactivity according to national guidelines.

Two recruitment methods were used. Sample 1 was recruited at sheltered housing sites in London, between November 2013 and January 2014. A housing trust, responsible for multiple London sheltered housing sites, permitted access to managers of local sites. Managers were informed of the study purpose and told that we were seeking older adults who sit for long periods and do little PA. Managers at five sites agreed to allow access to residents. Recruitment procedures at each site varied according to preferences of the site manager. At one site, the site manager gave a talk about the study to a group of potentially eligible participants, and those who were interested consented to participate at a subsequent face-to-face visit from the researcher. In the remaining four sites, managers suggested potential participants, based on the manager’s perceptions of their low PA and high SB, to the researcher, who approached them individually, explained the study and consented interested individuals. Sample 1 refusal rates were calculated by recording how many of those informed about the study (including potentially ineligible adults) did not wish to participate.

Sample 2 was recruited between March and June 2014, through written advertising materials and talks advertising the study at community and faith centres, and via a notice in an Age UK South London newsletter. Refusal rates could not therefore be calculated. Interested potential participants were pre-screened, by phone, using items from the International Physical Activity Questionnaire (IPAQ [42]), and a validated SB questionnaire [43] to establish their typical weekly MVPA and SB.

#### Data collection

Data were collected from Sample 1 between December 2013 and March 2014, and from Sample 2 between March and August 2014. Data collection took place at the participant’s home (Samples 1 and 2) or another location convenient to them (Sample 2). At the baseline session (Time 1; T1), participants completed a questionnaire of (quantitative) study measures, and then received the ‘On Your Feet to Earn Your Seat’ intervention booklet, together with nine tick-sheets to record adherence to the intervention tips^2^. The researcher explained to each participant the content and purpose of the booklet and tick-sheet, and how to complete the tick-sheet. Specifically, participants were told of the potentially independent health risks of SB and PA, and advised to follow the recommendations provided in the booklet on how to integrate more PA into everyday routines, while reducing SB. They were told that completing the tick-sheet could help them to monitor their progress in changing their PA and SB (and would enable the research team to monitor adherence to tips). No further advice or counselling was provided in either sample. Four and eight weeks post-baseline (T2 and T3 respectively), participants were visited again and asked to complete further quantitative measures. At T3, a face-to-face semi-structured interview was also conducted to capture participants’ views towards the intervention, and all tick-sheets were collected. T2 and T3 sessions were undertaken for measurement purposes only; no further active intervention was delivered, though participants were able to request clarifications of information provided to them at T1. Records were not kept of whether or what information was requested in this way.

Quantitative measures were self-administered where possible, or by the researcher at the participant’s request. All participants received a £10 (~US$15) shopping voucher at each of the three data collection points.

### Measures

#### Quantitative data

All quantitative data were self-reported. *Demographics* (gender, age, ethnicity, marital status, education) and *health status* variables were recorded at T1 only, for sample description purposes. Ethnicity was reported using UK census categories [44]. Marital status was reported using a single item (‘are you: single/married/widowed/divorced or separated?’). Education was recorded using two items, capturing the age at which participants left school, and whether they had completed a university degree (yes/no). Health status was assessed by a single item about long-term illness (‘have you any long-standing illness, disability or infirmity?’ [yes, please state/no]) [45].

*Sedentary behaviour* was assessed at T1-T3 using two measurements. One was an item derived from the IPAQ, assessing the total time spent sitting ‘while at home, when outdoors, or during leisure time’ (including ‘time spent sitting at a desk, visiting friends, reading, travelling on a bus, or sitting or lying down to watch television’) over the preceding seven days. IPAQ sitting items have been shown to have test-retest reliability, and to correlate with objectively measured inactivity [42, 46]. The second was the Measure of Older Adults’ Sedentary Time (MOST) [43], a multi-item questionnaire recording total time spent in seven common sedentary activities over the prior seven days (watching television, using the computer, reading, socializing, transportation, hobbies, ‘other activities’). The MOST has been validated against accelerometer step count readings, and shown to have test-retest reliability and be sensitive to changes in SB [43]. Data were summed across the seven activities, such that values denote total sitting time. MOST data were treated as missing where none of the seven items was completed, and eligible for analysis where at least one item was completed.

*Physical activity* was measured at T1-T3, using three single-items derived from the IPAQ relating to time spent walking, or in moderate or vigorous PA respectively, over the previous seven days (‘How much time in total did you spend [walking/doing vigorous physical activities/doing moderate physical activities] in the last 7 days?’). Vigorous activities were defined as those ‘that take hard physical effort and make you breathe much harder than normal’, and moderate as those ‘that take moderate physical effort and make you breathe somewhat harder than normal’. For both items, participants were asked to consider only ‘those physical activities that you did for at least 10 min at a time’. IPAQ PA items have been shown to have test-retest reliability, and to correspond with multiple objectively measured PA indicators [42, 46]. Responses to all IPAQ SB and PA items were provided in hours and minutes, and converted to total minutes for the purpose of analysis.

SB and PA *habit* were each measured using the Self-Report Behavioural Automaticity Index (SRBAI) [47], a four-item subscale of the Self-Report Habit Index (SRHI) [48]. The SRBAI focuses on the automaticity that characterises habitual responses [29, 49]. Both the SRHI and SRBAI show sensitivity to theorised effects of habit on action, and convergence with implicit association-based habit measures [47]. A systematic re-analysis of previous SRHI applications showed the SRBAI to have consistently strong internal reliability, and convergent validity with its parent index [47]. SRHI/SRBAI applications with greater contextual specificity are likely to minimize respondent interpretation error [50], and so item wordings specified a behaviour (‘sitting…’) and a context (‘…during my leisure time’). For SB habit, items followed the stem ‘Sitting during my leisure time is something…’, and PA habit items followed the stem ‘Physical activity during my leisure time is something…’ (‘…I do automatically’, ‘…I do without thinking’, ‘…I do without having to consciously remember’, ‘…I start doing before I realize I’m doing it’; 1 = strongly disagree, 7 = strongly agree). Mean scores were generated for each index, with higher scores indicating stronger habit. Reliability was good at all time points (SB habit, α range Sample 1: .77-.95, Sample 2: .88-.96; PA habit, α range Sample 1: .86-.97, Sample 2: .86-.95).

*Adherence to tips* was assessed via 7-day tick-sheets. Participants were asked to record a tick on each day on which they completed each recommended activity, for the study duration. For one tip, which recommended setting a manageable walking target (see Additional file 1: Table S1), participants were asked to record their daily target and whether it had been achieved.

#### Qualitative data

Semi-structured interviews covered five topics: experiences of using the leaflet, barriers to adherence, habit-formation, whether further support was required, and suggestions for improvement. Participants’ responses shaped progression through topics. Audio recordings of interviews were transcribed verbatim.

### Analysis

#### Quantitative data

Refusal rates (Sample 1 only) and attrition rates were summarized using descriptive statistics. Rates of adherence to each tip were calculated using the seven tick-sheets for which full (7-day) data were available (i.e., Weeks 2–8; see Endnote 2). Weekly adherence to each tip was calculated by summing, for each tip, the number of ticks recorded in that week and dividing the total by seven (i.e. 7 days). Mean total adherence to each tip was calculated by summing all ticks for each tip across all seven tick-sheets and dividing by 49 (i.e. 7 days × 7 weeks). Global mean total adherence across all tips was calculated by summing the mean total adherence to each of the 16 tips and dividing by 16. A supplementary analysis was undertaken of weekly adherence across all tips, as calculated by summing all ticks for all tips in each week and dividing by 112 (i.e. 16 tips × 7 days). All rates were multiplied by 100, to allow expression as percentages.

The purpose of reporting SB and PA behaviour and habit strength across the three timepoints was to investigate behavioural responding as an indicator of acceptability, with trends towards decreased SB and/or increased PA, regardless of statistical significance, seen to reflect intervention acceptability. Nonetheless, inferential statistical tests were run, and p values reported, for completeness. Behaviour and habit changes were tested using repeated-measures ANOVA. Where normality and equality of variance assumptions were not met, Friedman’s two-way ANOVA for non-parametric data were used. A supplementary analysis was run to document the number of participants showing changes from baseline, at either follow-up point, on SB and PA behaviour and habit indices. Quantitative data were assessed for each sample in isolation, and, with the exception of attrition analyses, run only for those who completed all three study timepoints.

Quantitative analyses were run to describe trends observed in available data, rather than to investigate intervention effects. Thus, missing data were handled using pairwise deletion for descriptive purposes, and listwise deletion for inferential statistical tests.

#### Qualitative data

Interview transcripts were analysed by two coders, using thematic analysis [51]. Themes were inductively developed and iteratively refined by one coder, and verified through discussions with a second coder. Disagreements were resolved through discussion between coders. Only excerpts relating to intervention content were eligible for analysis; responses relating to visual presentation were used to refine materials, but are not presented here. Although thematic analysis is not well-suited to formal comparisons between groups, coders did not observe differences between the two samples in the content or fit of each theme, and so analyses are reported for both samples combined.

## Results

### Sample description and attrition

#### Sample 1

**Fig. 1:**
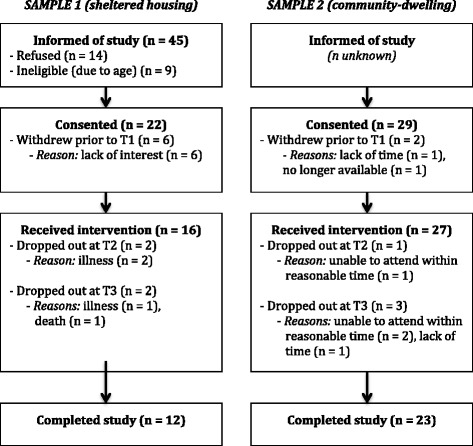
Participant flow, Samples 1 and 2

Of 45 older adults notified of the study, 14 refused to participate (31 %), and nine (20 %) were ineligible due to age (see Fig. 1)^3^. Twenty-two participants were consented, though six withdrew prior to T1, citing lack of interest.

Of 16 participants who received the intervention, 12 (75 %) completed the study. Two participants (13 %) withdrew prior to T2 due to illness, and two (13 %) dropped out between T2 and T3, one due to illness and another to death. All of these adverse events were unrelated to study participation. All participants who dropped out after T1 were male, with longstanding illness, and without university education. While statistical comparisons were not undertaken due to small samples, non-completers tended to spend less time walking or in moderate PA, and spent more time sitting than did completers (see Table 1).

**Table 1:** Baseline demographics, physical activity and sedentary behaviour of completers vs non-completers, Samples 1 and 2

		Sample 1		Sample 2	
Participant characteristics		Completers (*n* = 12)	Non-completers (*n* = 4)	Completers (*n* = 23)	Non-completers (*n* = 4)
Demographics					
Gender	*n*	12	4	23	4
	Male	10 (83 %)	4 (100 %)	7 (30 %)	2 (50 %)
	Female	2 (17 %)	0	16 (70 %)	2 (50 %)
Age	*n*	12	4	23	4
	Mean (SD)	66.42 (4.81)	69.25 (4.50)	66.91 (4.18)	69.50 (6.85)
Ethnicity	*n*	11	4	23	4
	White	9 (82 %)	2 (50 %)	11 (48 %)	1 (25 %)
	Black	1 (9 %)	0	8 (35 %)	3 (75 %)
	Asian	1 (9 %)	0	3 (13 %)	0
	Mixed or other	0	2 (50 %	1 (4 %)	0
Marital status	*n*	12	4	23	4
	Single	8 (67 %)	2 (50 %)	3 (13 %)	2 (50 %)
	Married	0	1 (25 %)	11 (48 %)	1 (25 %)
	Widowed	1 (8 %)	0	5 (22 %)	0
	Divorced or separated	3 (25 %)	1 (25 %)	4 (17 %)	1 (25 %)
Longstanding illness	*n*	11	4	22	4
	Yes	8 (67 %)	4 (100 %)	16 (70 %)	3 (75 %)
	No	3 (33 %)	0	6 (26 %)	1 (25 %)
Education: university	*n*	11	4	26	4
	Yes	0	0	11 (48 %)	1 (25 %)
	No	11 (100 %)	4 (100 %)	12 (52 %)	3 (75 %)
Education: age leaving school	*n*	12	4	22	4
	Mean (SD)	15.27 (1.84)	15.00 (3.74)	17.41 (2.94)	16.75 (2.75)
Sedentary behaviour					
Sitting time (IPAQ), mins/week	*n*	10	4	23	4
	Mean (SD)	2082.00 (1614.26)	4095.00 (1547.93)	2695.43 (1041.47)	960.00 (1416.47)
Sitting time (MOST), mins/week	*n*	11	4	23	4
	Mean (SD)	2445.83 (2474.71)	3825.00 (2069.27)	3534.13 (1895.25)	3412.50 (1643.60)
SB habit	*n*	11	4	22	4
	Mean (SD)	4.00 (0.72)	4.50 (0.57)	3.80 (0.87)	3.50 (0.88)
Physical activity					
Walking, mins/week	*n*	12	4	23	4
	Mean (SD)	236.67 (384.72)	70.00 (49.66)	341.74 (476.17)	705.00 (644.17)
Moderate PA, mins/week	*n*	12	4	23	4
	Mean (SD)	48.33 (121.34)	15.00 (30.00)	143.48 (396.03)	37.50 (56.78)
Vigorous PA, mins/week	*n*	12	4	26	4
	Mean (SD)	20.00 (53.26)	0	75.13 (130.30)	0
PA habit	*n*	11	4	23	4
	Mean (SD)	3.27 (0.99)	2.93 (1.19)	2.89 (0.96)	2.50 (1.00)

‘Non-completers’ were participants who completed T1 but withdrew prior to T3. Ns differ due to missing data. PA = Physical activity, SB = Sedentary behaviour, SD = Standard deviation

As Table 1 shows, among Study 1 completers, mean sitting time reported in response to the IPAQ was 2082.0mins/week, and in response to the MOST 2445.8mins/week. Mean walking time was 236.7mins/week, mean moderate PA 48.3mins/week, and mean vigorous PA 20.0mins/week. Mean SB habit was at the midpoint (mean 4.00), and PA habit below the midpoint (mean 3.27).

#### Sample 2

Twenty-nine participants were consented, of whom two (7 %) withdrew prior to T1, one citing lack of time for participation and the other having had to leave the country unexpectedly (Fig. 1). Of 27 participants who received the intervention, 23 (85 %) completed the study. One (3 %) withdrew prior to T2, and three (11 %) between T2 and T3, because they were unable to attend within two weeks of the due dates (two participants), or reported a lack of time for participation (one participant). Most of the four non-completers had longstanding illnesses (3 participants; 75 %), or no university education (3; 75 %), and none reported vigorous PA over the preceding week. Non-completers spent more time walking, less time in moderate PA, and less time sitting than did completers. The magnitude of between-group sitting time differences varied considerably across sitting time indices (mean difference [completers – non-completers], IPAQ = 1735mins; mean difference [completers – non-completers], MOST = 122mins; see Table 1).

As Table 1 shows, mean sitting time reported in response to the IPAQ was 2695.4mins/week, and in response to the MOST 3534.1mins/week. Mean walking time was 341.7mins/week, mean moderate PA 143.5mins/week, mean vigorous PA 75.1mins/week. Mean SB habit was at the midpoint (mean 3.80), and PA habit was below the midpoint (mean 2.89).

### Intervention adherence

#### Sample 1

Eleven participants (92 %) returned at least eight tick-sheets, and one participant did not return any, stating that they had been lost. Global mean adherence was 40.48 %. As Table 2 shows, mean total adherence rates were highest for Tip 2 (‘make ad breaks active’; 60.85 %, range 45.45 % to 67.53 %) and lowest for Tip 5 (‘tiptoe through the queue’; 14.84 %, mean per-week adherence range 6.49 % to 35.06 %). (See Additional file 2: Table S2 for week-by-week per-tip adherence rates.) Across all tips, highest adherence was observed between weeks 2 and 4, with the exception of Tips 9c (‘toe rises’; weeks 2 and 6) and 9 g (‘lift a tin of food in each hand’; week 7). Lowest adherence for all tips was observed at weeks 7 or 8. (See Additional file 3: Table S3 for weekly adherence rates across all tips.) Mean adherence was above 50 % for five of the sixteen tips (Tips 1, 2, 9a, 9b, 10), indicating these were typically more often enacted than not.

**Table 2:** Mean total adherence to intervention tips, Weeks 2–8, Samples 1 and 2, completers only

Tips	Adherence							
	Sample 1				Sample 2			
	N	Mean (SD)	Lowest weekly mean adherence	Highest weekly mean adherence	N	Mean (SD)	Lowest weekly mean adherence	Highest weekly mean adherence
“1. Leave the house daily: Ensure that you go out at least once a day.”	Weeks (W) 2–7, Ns = 11; W8, N = 10	55.31 % (35.65)	40.00 % (W8)	59.74 % (W2,W4)	W2-5, N = 22; W6-8, N = 21	81.63 % (21.03)	71.43 % (W8)	85.71 % (W4)
“2. Make ad breaks active: When you watch TV, stand up or walk around during breaks between programmes.”	All weeks: N = 11	60.85 % (41.16)	45.45 % (W8)	67.53 % (W2)	W2-5, N = 21; W6-8, N = 20	62.96 % (33.27)	50.71 % (W8)	71.43 % (W2)
“3. Take a stand: Stand up when waiting for a bus or train.”	All weeks: N = 11	38.78 % (41.48)	27.27 % (W8)	44.16 % (W3)	W2-5, N = 22; W6-8, N = 21	64.24 % (35.37)	58.44 % (W5)	67.53 % (W3)
“4. Time to stretch: If you are using a computer, set an alarm to go off every 20 min. When it rings, stand up and stretch.”	All weeks: N = 11	43.23 % (42.34)	23.38 % (W8)	64.94 % (W2)	W2-5, N = 22; W6-8, N = 21	62.20 % (36.74)	52.38 % (W8)	64.29 % (W4)
“5. Tiptoe through the queue: When waiting in a queue … stand on your tip toes and then drop back down onto your heels gently.”	All weeks: N = 11	14.84 % (19.98)	6.49 % (W7)	35.06 % (W2)	W2-5, N = 22; W6-8, N = 21	38.97 % (33.18)	31.97 % (W6)	42.21 % (W3)
“6. Watch your step: Set a target of walking at least 1500 steps each day.”	All weeks: N = 11	40.45 % (40.99)	19.48 % (W8)	50.65 % (W4)	W2-5, N = 22; W6-8, N = 21	48.40 % (40.78)	45.45 % (W5)	51.02 % (W8)
“7. Sit to stand with no hands: Each time you stand up, try doing it without using your hands.”	All weeks: N = 11	49.72 % (39.51)	29.87 % (W8)	61.04 % (W3)	W2-5, N = 22; W6-8, N = 21	72.50 % (29.20)	62.99 % (W5)	83.77 % (W3)
“8. Improve your posture: Stand with your back to the wall with your heels two inches from it … and move the back of your head towards the wall.”	All weeks: N = 11	37.66 % (39.07)	27.27 % (W8)	42.86 % (W3)	W2-5, N = 22; W6-8, N = 21	55.00 % (35.62)	48.30 % (W8)	64.94 % (W4)
“9. Limber up:								
9a. Calf stretch	All weeks: N = 11	52.32 % (41.22)	41.56 % (W8)	61.04 % (W4)	W2-5, N = 22; W6-8, N = 21	57.14 % (40.40)	49.66 % (W6)	66.88 % (W2)
9b. Chest stretch	All weeks: N = 11	54.73 % (40.03)	40.26 % (W8)	62.34 % (W2)	W2-5, N = 22; W6-8, N = 21	60.16 % (33.66)	54.42 % (W8)	69.48 % (W4)
9c. Toe rises	All weeks: N = 11	47.31 % (41.49)	23.38 % (W8)	55.84 % (W2, W6)	W2-5, N = 22; W6-8, N = 21	61.90 % (32.85)	58.50 % (W6)	69.48 % (W2)
9d. Walk as if on a tightrope across the floor	All weeks: N = 11	35.81 % (39.48)	18.18 % (W8)	45.45 % (W4)	W2-5, N = 22; W6-8, N = 21	46.16 % (36.35)	37.41 % (W8)	59.74 % (W3)
9e. March on the spot	All weeks: N = 11	39.33 % (40.26)	15.58 % (W8)	51.95 % (W2)	W2-5, N = 22; W6-8, N = 21	53.26 % (37.18)	44.90 % (W8)	66.88 % (W3)
9f. Walk your fingers up the wall	All weeks: N = 11	32.47 % (37.37)	12.99 % (W8)	48.05 % (W2)	W2-5, N = 22; W6-8, N = 21	46.26 % (37.04)	36.73 % (W8)	57.79 % (W2)
9g. Lift a tin of food in each hand.”	All weeks: N = 11	43.78 % (42.92)	18.18 % (W8)	53.25 % (W7)	W2-5, N = 22; W6-8, N = 21	38.97 % (36.47)	31.29 % (W8)	48.05 % (W4)
“10. Wall push-ups: do 10-push ups against a wall each morning.”	All weeks: N = 11	50.28 % (43.26)	28.57 % (W8)	62.34 % (W3)	W2-5, N = 22; W6-8, N = 21	58.89 % (33.05)	50.34 % (W8)	68.83 % (W2)

SD = Standard deviation, W = week number

#### Sample 2

Twenty-two participants (92 %) returned at least eight tick-sheets. One participant returned only five tick-sheets, and one did not return any tick-sheets. Global mean adherence was 57.86 %. Mean total adherence rates were lowest for Tip 5 (‘tiptoe through the queue’; 38.97 %, range 31.97 % to 42.21 %), and highest for Tip 1 (‘leave the house daily’; 81.63 %, range 71.43 % to 85.71 %). Across all tips, highest adherence was typically observed between weeks 2 and 4, and lowest adherence between weeks 5 and 8, most typically at week 8. Mean adherence rates were above 50 % for eleven tips (Tips 1, 2, 3, 4, 7, 8, 9a, 9b, 9c, 9e, 10).

### Behavioural responses to intervention

#### Sample 1

As Table 3 indicates, sitting time measured by the IPAQ tended to increase between the three study timepoints. and measured using the MOST, was higher at T2 and T3 than at T1. Walking increased from T1 to subsequent timepoints, though dipped notably between T2 and T3. Moderate PA decreased between the three timepoints. Vigorous PA decreased between T1 and T2, but increased markedly between T2 and T3. There were no clear patterns of change in mean SB or PA habit scores. (See Additional file 4: Table S4 for analysis of the number of participants increasing or decreasing SB or PA habit or behaviour.)

**Table 3:** Physical activity, sedentary behaviour and habit at T1-T3, Samples 1 and 2, completers only

	Sample 1*				Sample 2*			
	T1 (mean, SD)	T2 (mean, SD)	T3 (mean, SD)	p for trend	T1 (mean, SD)	T2 (mean, SD)	T3 (mean, SD)	p for trend
Sedentary behaviour								
Sitting time (IPAQ), mins/week	2082.00 (1614.26)	2280.00 (1337.41)	2422.50 (1253.30)	.76	2695.43 (1041.47)	1841.52 (912.67)	1639.57 (861.57)	<.001
Sitting time (MOST), mins/week	2445.83 (2474.71)	4145.25 (3143.73)	3011.42 (2496.47)	.33	3534.13 (1895.25)	3089.13 (1317.22)	2530.43 (1416.67)	.047
Sitting habit**	4.00 (0.72)	3.83 (0.71)	3.96 (0.86)	.89	3.80 (0.87)	3.77 (0.61)	3.49 (0.93)	.11
Physical activity								
Walking, mins/week	236.67 (384.72)	633.33 (1086.29)	292.50 (351.78)	.18	341.74 (476.17)	386.96 (470.46)	485.65 (521.42)	.003
Moderate PA, mins/week	48.33 (121.34)	39.17 (67.08)	34.67 (72.48)	.31	143.48 (396.03)	90.43 (134.65)	145.22 (204.39)	.22
Vigorous PA, mins/week	20.00 (53.26)	15.00 (51.96)	71.67 (242.03)	.36	75.13 (130.30)	166.96 (291.82)	177.83 (271.56)	.06
PA habit**	3.27 (0.99)	2.98 (0.84)	3.65 (0.82)	.14	2.89 (0.96)	3.47 (0.76)	3.47 (0.84)	.26

*Sample 1 N = 12 for all variables except T1 sitting time (IPAQ) (N = 10), T2 sitting time (IPAQ) (N = 11), T1 sitting time (MOST) (N = 11), T1 sitting habit (N = 11), and T1 PA habit (N = 11). Sample 2 N = 23 for all variables except sitting habit (N = 22). Tests of trends based on listwise deletion. ** Habit measured on a 1–7 scale, where 1 = weak or no habit, and 7 = strongest habit

#### Sample 2

Sitting time (measured by both IPAQ and MOST), and SB habit, decreased between the three timepoints. Walking and vigorous PA increased between the three timepoints. PA habit increased between T1 and T2, and remained stable at T3. Moderate PA dropped between T1 and T2, but increased markedly between T2 and T3, to around T1 level. (See too Additional file 4: Table S4.)

### Qualitative responses to intervention

Three acceptability-related themes were extracted: the intervention as a spur to action; gains in behaviour, habit, health, and wellbeing; and psychological, physical and social barriers to intervention use. Unless otherwise stated, all points apply to participants from both samples.

#### Intervention as a spur to action

The intervention was mostly viewed positively. For many, the leaflet provided new information about the health risks of SB, and raised awareness of participants’ own sedentary behaviour:

Sample 2, participant 13 (S2, P13): *It made me conscious I couldn’t sit for hours in one place doing nothing.*

S2, P6: *I realized how, if you have a quiet day and you don’t go out, how much less you move around than you [otherwise would] do.*

Tips were deemed easy to follow and enact:

S2, P7: *It’s so easy to understand and they even show you [how to do them] – if you found that difficult to do, I think you need more than exercise!*

Several of the tips were novel to many participants.

S2, P13: *Calf stretch [was] my favourite tip; I didn’t know I could exercise my feet like that.*

Other tips served as reminders of forgotten activities previously recommended to them. The focus on making ‘small changes’ to PA prompted some participants to reconsider what ‘counts’ as PA, and several reported becoming more aware of everyday PA opportunities afforded by the environment:

S1, P10: *I didn’t realize that [small changes can be beneficial] before, but I do now. Plus they’re things that you can do while you are at home. You can just sit there watching television and do some of them.*

Some participants in Sample 2 used the booklet or tick-sheet as environmental triggers to PA:

S2, P7: *When I get up in the morning, [the booklet] is the first thing I see. I bring in my breakfast and when I’m finished … I will do the exercise.*

For some, researcher visits were an important intervention component:

S2, P10: *Even only knowing that you would come [to visit me] and having to tick the boxes inspires you to do more activities.*

#### Gains in behaviour, habit, health and wellbeing

Several participants in Sample 2 adopted new personal rules for incorporating PA into everyday settings.

S2, P12: *I used to go shopping 3 times a week but now I do the shopping for the whole week so I can carry the bags.*

Many reported performance of the tips having become more habitual:

S2, P10: *After a while you just do it, it becomes automatic. It becomes like saying your prayers every day, you don’t need to remind yourself.*

Activities that were seen to be less easy to incorporate into everyday patterns were less likely to be enacted and so unlikely to become habitual:

S2, P6: *Finger walking – it’s not something [you can] build into your daily life.*

One participant reported gains in positive affect, confidence and wellbeing:

S2, P1: *I can follow this programme and keep myself as mobile or as physically able as possible. It gives me hope and encouragement, and that makes me happy.*

Participants attributed health benefits to the intervention, including reductions in pain, stiffness (one participant, Sample 2), increased energy levels (one participant, Sample 2), and enhanced sleep quality (one participant, Sample 1):

S1, P7: *When I do the exercises I feel a bit tired, and then by the evening … I go to sleep. I do sleep well. Before I couldn’t [sleep], I used to twist and turn, had aches and pains … but [since] then it’s improved.*

*Physical, psychological and social barriers to intervention use.* Several participants reported that they were unable to perform some tips due to pre-existing health conditions (e.g. arthritis, knee pain, hip problems), though some were able to adapt tips to their personal circumstances (e.g. using lighter food tins to lift weights).

Some felt the booklet provided insufficient health justification for the tips, which acted as a psychological barrier:

S2, P1: *At first I didn’t quite realize why we’re doing one or two of the exercises. … You told me at your second visit [that] it was for my balance. When I knew it was for my balance, I thought, ‘oh yes, I mustn’t forget that one, I must do it’.*

A failure to identify with the target group implied by the booklet text or images, cited among participants in Sample 2, may have limited engagement:

S2, P13: *I saw the pictures and I saw images of people in the leaflet and I was saying, is this really for me? […] The photographs made me think it might not be for me … there are no men there, only women, and only of a certain age. […] I don’t see myself as very inactive.*

Fear of embarrassment acted as a barrier to enacting some tips in public settings:

S1, P10: *If you do it while standing at a bus stop, you’ll get strange looks by people if you are standing there. They’ll think, “what’s the matter with him?”*

## Discussion

This uncontrolled trial explored the acceptability of an intervention to displace SB with light intensity PA in insufficiently active and sedentary older adults (‘On Your Feet to Earn Your Seat’). Four sources of information were used: rates of attrition and adherence to intervention recommendations, pre-post behavioural responses, and reflections on the intervention expressed in semi-structured interviews. Among a sample living in warden-assisted accommodation (Sample 1), attrition was 25 %, mean adherence was around 40 %, and no clear changes were observed in sitting or physical activity time or habit. Refusal among those informed about the intervention was 45 %. Among independently-living older adults (Sample 2), attrition was 15 %, mean adherence was 58 %, and trends were observed towards decreased sitting time, and increases in walking, moderate PA, and PA habit. Qualitative data indicated that participants in both samples were favourable towards the intervention, and reported activity gains and benefits to health and wellbeing. The intervention appeared generally acceptable to both samples.

Our results testify to the acceptability of reducing SB, and using the habit-formation model as a basis for doing so, among older adults [21, 23]. Attrition rates were broadly similar to those observed in previous PA intervention trials conducted among older adults. Median drop-out across 22 studies synthesised by King and colleagues [52] was 17 %, albeit over longer periods (median 6 months) than the current 8-week study. While longer-term follow-up periods are needed, with larger samples, our data tentatively suggest, based on attrition, that our intervention is roughly as acceptable to previous PA promotion interventions for older adults. Participants found the intervention motivating and tips easy to understand and follow, and many reported the recommended behaviours becoming automatic (i.e., habitual). While the use of habit theory as an intervention basis is novel [29], some of the specific behavioural recommendations were selected because they have previously been shown to be effective in reducing SB, such as standing during TV commercial breaks [22, 53]. Although collected primarily to inform the next stage of our intervention development project [25], to our knowledge our data offer the first evaluation of the acceptability of practical suggestions for SB reduction, and highlight potential barriers to uptake. In particular, strategies that focus on incorporating PA into social situations may fail where the recommended activity is non-normative: a tip recommending standing on tiptoes while queuing was poorly adhered-to, and attracted most negative feedback in interviews due to anticipated embarrassment. Tips thought to be inadequately justified were also viewed negatively by some participants. These findings point to the importance of pursuing strategies that are not only likely to yield greatest SB reductions if followed, but also likely to be understood and implemented by the target population [34].

Surprisingly, while many participants spontaneously reported behaviours becoming automatic, adherence rates generally declined over the 8-week study period, in both samples. This appears to conflict with predictions from habit theory that, as habit becomes stronger, the habitual behaviour is likely to be more frequently enacted [54]. This finding may however reflect declines in use of the intervention tick-sheets to record adherence, rather than reductions in true adherence. While we entered into analysis all participants who completed at least one ticksheet, five participants (three in Sample 1, two in Sample 2) returned blank ticksheets at the final week. It is not possible to discern true declines in adherence to tips from reductions in using tick-sheets. While we asked participants to complete tick-sheets for both intervention and data collection purposes, it is feasible that, as habit formed, participants became less dependent on consciously regulating their behaviour through self-monitoring, and so were less inclined to complete the tick-sheets. Indeed, one longitudinal study of regular recycling showed that, as habit formed, participants became less dependent on reminders, another form of self-regulatory aid [55].

Nonetheless, in response to declining adherence, and other indicators of potentially suboptimal acceptability, four amendments have been made to the intervention, prior to its evaluation in a controlled trial [25]. First, the recommended setting for tiptoeing has been changed from queuing in public to the kitchen sink, so promoting private performance which avoids potential social discomfort, while retaining the context-dependency necessary for habit to form [32]. Second, additional justification has been provided for incremental PA gains, based on explaining the dose-response relationship between PA and health [56]. Third, the intervention now explicitly recommends placing the booklet in a prominent place so it may act as a visual cue to PA, which was reportedly helpful for some participants in Sample 2. Lastly, in light of declining adherence, the intervention delivery protocol has been amended to offer additional motivational support and advice in a phone call to intervention recipients at four weeks post-baseline [25].

We sought to recruit two samples of sedentary older adults: Sample 1 comprised residents of sheltered housing sites, where PA levels are generally low [41], and Sample 2 comprised members of the general public who did not meet national PA recommendations (<150mins/week MVPA). Descriptive statistics confirmed that, at baseline, Sample 1 was less physically active than Sample 2, and both groups self-reported accruing at least 5 h of daily sitting at baseline. Yet, nearly half of those approached for Sample 1 refused to participate, and those who did participate were more likely to drop out of the study and less likely to adhere than Sample 2, and did not appear to change their behaviour in response to the intervention. Although Sample 1 attrition was attributable to unrelated health problems, higher refusal and lower adherence rates relative to Sample 2 suggest our intervention may be less acceptable to those most in need of increasing their PA, given baseline levels of activity. Our data do not reveal why this may be, but several explanations are plausible. We purposefully recruited Sample 1 from sheltered housing, because residents were likely to be both sedentary and highly inactive [41], an assumption borne out by self-reported low PA and high SB at baseline. Yet, relative to the general UK older population, UK sheltered housing residents tend to have poorer physical and mental health and functioning [57, 58], which may limit PA. Indeed, our participants frequently cited poor health as a barrier to intervention engagement. Our intervention may therefore have been less well suited to the needs of Sample 1. Acceptability may also have been lowered because Sample 1 participated during Winter, whereas Sample 2 participated in Spring and Summer. While most tips could be performed indoors or outdoors, three required performance outside the home (‘leave the house daily’, ‘take a stand’, ‘tiptoe through the queue’; see Table 2). Cold weather can act as a barrier to PA, especially among older people [59, 60]. PA promotion may therefore naturally be less acceptable during colder months. Between-sample differences may also reflect gender effects: Sample 1 was mostly male, and Sample 2 predominantly female. Of those aged 65 and above, females tend to be less physically active than males [61] and so may have been more receptive to PA promotion.

Alternatively, selection biases may have increased acceptability within Sample 2. Whereas Sample 1 was recruited using outreach methods (i.e. face-to-face recruitment visits within their homes), Sample 2 was approached via advertising materials that required those interested to contact the researcher. Sample 2 may have been more socially active, which has been associated with greater PA [62], and more open to PA promotion. It is also possible that our evaluation methods were less suited to Sample 1. Respondents often find behaviour and habit questionnaires confusing and difficult to complete [50, 63]. Sample 1 was less educated than Sample 2, and may have had less experience and confidence in reflecting on their behaviour, rendering responses unreliable. Moreover, the lack of change on quantitative behaviour indices conflicted with the positive behavioural and health impacts reported by many participants in Sample 1 in interviews. Acceptability among Sample 1 may therefore have been underestimated by the quantitative measures employed.

Study limitations must be acknowledged. We recruited two samples of sedentary older adults, to represent varying degrees of inactivity, under the assumption that sheltered housing residents (Sample 1) would be more sedentary and less active than independently-living older adults (Sample 2). Yet, mean reported baseline sitting time was lower among Sample 1 than Sample 2. Additionally, both samples were reportedly relatively physically active, reporting several hours of walking each week. These data may reflect true behavioural patterns. At four of the five recruitment sites, participants were recruited to Sample 1 by site managers, on the basis that managers believed that participants spent little time in PA and much time in SB. Managers’ perceptions may however have underestimated true PA and overestimated SB. Our recruitment strategy may have failed to capture the sedentary and inactive sheltered housing residents who may benefit most from an SB reduction intervention. We agreed to delegate recruitment to managers as a condition for access to residents, but this may have biased participant selection. Another possibility is that lower SB in Sample 1 than Sample 2 reflected more time spent sleeping, which does not qualify as SB and was not measured in this study. Between-sample differences may alternatively reflect inaccurate responding: self-reports generally overestimate their PA and underestimate SB [61, 64]. One study showed that only 10 % of males and 8 % of females who reported doing at least 150 min of moderate-to-vigorous PA were found to have done so when their PA was measured by an accelerometer [61]. The involvement of site managers in recruitment may perhaps have increased socially desirable responding in Sample 1, possibly leading to further under-reporting of true SB and over-reporting true PA. Without objective data for validation purposes, the accuracy of self-report cannot be explored, and it is unclear whether Sample 1 were any more inaccurate than Sample 2 in recalling their sitting time. Interestingly, both groups gave markedly different self-reports of baseline sitting in response to a single IPAQ-derived item versus a multi-item scale (the MOST) that sums sitting time across activities (discrepancies of 364 and 839 min for Samples 1 and 2 respectively), suggesting inconsistent responding. Supplementary analysis of the magnitude of changes in habit and behaviour questioned the validity of some self-reports, with large fluctuations observed in SB and PA across study timepoints. While such fluctuations may of course reflect actual behaviour changes, some change scores appeared unrealistic, especially in response to the MOST, with one participant reporting a decrease between baseline and 4-week follow-up of 6300 weekly SB minutes, equivalent to 15 daily hours. Such problems may reflect the inherent measurement challenge posed by light PA and sitting time, instances of which are typically unremarkable, unmemorable and so potentially difficult to self-report [65]. This challenge may be compounded by interpretation errors: a ‘think-aloud’ study of older adults revealed misunderstandings of self-report sitting items, and erroneous inferences of actual sitting time from typical daily time spent in salient seated activities [66].

SB and PA data were described only for study completers. Yet, nearly half of those invited to join Sample 1 refused to receive the intervention, and 25 % of intervention recipients in Sample 1 and 15 % in Sample 2 dropped out prior to completion. Non-completers tended to have poorer health and be less active than completers. Thus, observed SB and PA patterns may not reliably reflect effectiveness. Indeed, the reliance on self-report measures, alongside the absence of a control group, and small sample size, means that, even among study completers, observed patterns of SB and PA are tentative and cannot be attributed to the intervention. However, the aim of this study was not to draw conclusions about intervention effectiveness, but rather to use behavioural data as one of several indices of acceptability, on the assumption that positive behaviour change can be taken to indicate that potential participants are willing to respond positively to the intervention. Our ongoing trial uses more rigorous intervention evaluation methods, by employing a treatment-as-usual control group, thigh-worn activPAL accelerometers to better assess changes in SB and PA and estimate effects attributable to the intervention, and an intention-to-treat analysis to account for non-completers [25].

## Conclusions

Results from this small early-phase study indicate that a low-cost and largely self-administered, habit-based intervention to displace SB with PA was generally acceptable among sedentary and insufficiently active older adults. Four main changes have been made to the intervention following the findings from this study. A controlled trial of the next iteration of the intervention is underway [26].

## Additional files

Additional file 1: Table S1. Intervention content: description and component behaviour change techniques. Additional file 2: Table S2. Mean total per-week adherence to intervention tips, Weeks 2–8, Samples 1 and 2, completers only. Additional file 3: Table S3. Mean and median weekly adherence across all tips, Weeks 2–8, Samples 1 and 2, completers only. Additional file 4: Table S4. Changes in physical activity, sedentary behaviour and habit from T1, Samples 1 and 2, completers only.

## Abbreviations

M: Mean
MOST: Measure of Older Adults’ Sedentary Time
MVPA: Moderate-to-vigorous physical activity
PA: Physical activity
SB: Sedentary behaviour
SD: Standard deviation
SRBAI: Self-Report Behavioural Automaticity Index
SRHI: Self-Report Habit Index
TV: Television
W: Week

## Footnotes

1 Age criteria were imposed because the intervention was designed for those aged 60–75 years. Nonetheless, those aged over 75 years with sufficient physical capability could potentially benefit from our intervention. Thus, to increase the amount of feedback received on the intervention, people who were interested in the study but did not meet the age criteria (*n* = 9; one aged 58, 8 aged over 75 years) were nonetheless recruited. Of these, two dropped out between baseline (T1) and 4-week follow-up (T2), one due to illness and one due to a fall, both self-reportedly unrelated to the study. Seven participants completed the study. Given that the intervention is designed for 60–75 year olds in particular, these participants are excluded from the present analysis.

2 Each ‘tick-sheet’ related to a Monday-Sunday period. Nine tick-sheets were given to participants to ensure those who began the intervention on days other than Monday were able to monitor their behaviour for at least eight weeks (56 days). To minimize confusion, ineligible days on the first tick-sheet were manually blocked out by the researcher.

3 When including the 9 ineligible participants who were consented to the study (see Endnote 1), refusal and attrition rates were as follows. Of 45 older adults notified of the study, 14 refused to participate (31%), and 31 (69%) were consented, of whom six withdrew prior to T1, citing lack of interest. Of the 25 participants who received the intervention, 19 (76%) completed the study. Four participants (16%) withdrew prior to T2, three due to illness, and one due to a fall. Two (8%) dropped out between T2 and T3, one due to illness and another to death.
