# Supplementary material for: Acceptability of a theory-based sedentary behaviour reduction intervention for older adults (‘On Your Feet to Earn Your Seat’)
Source: BMC Public Health. 2015 Jul 2;15:606. doi: 10.1186/s12889-015-1921-0 (PMC4489366; doi:10.1186/s12889-015-1921-0)
Supplement: Additional file 1: Table S1. — Intervention content: description and component behaviour change techniques. [file 12889_2015_1921_MOESM1_ESM.doc]

**Additional file 1: Table S1.** Intervention content: description and component behaviour change techniques.

| *Booklet section* | *Informational content / behaviour change recommendations ** | *Specific PA forms targeted in tips* | *Behaviour change techniques* |
| --- | --- | --- | --- |
| Motivational text | - Regular PA in older age protects physical and mental health.  - PA includes aerobic, stretching, balance and strengthening.  - Sitting time is a risk factor for physical health.  - Limiting sitting to 20mins may protect health.  - Context-dependent repetition forms habit, which can maintain behaviour. | N/A | Information on health consequences; Framing/reframing;  Habit formation |
| Tips | “1. Leave the house daily: Ensure that you go out at least once a day. […] Don’t hesitate to use a stick if you need to.” | No explicit PA form, but conducive to standing (balance) and walking (aerobic) | Action planning;  Goal setting (behaviour);  Adding objects to the environment |
|  | “2. Make ad breaks active: When you watch TV, stand up or walk around during breaks between programmes. […] Try to watch TV for no more than one hour at a time, including two active breaks. Leave the remote control by the TV so that you have to get up to change channel.” | Standing (balance)  Walking (aerobic) | Prompts/cues;  Goal setting (behaviour);  Restructuring the physical environment;  Habit formation |
|  | “3. Take a stand: Stand up when waiting for a bus or train. Stay standing as long as possible. […] Make the sight of an empty seat a reminder to stand up.” | Standing (balance) | Prompts/cues;  Framing/reframing;  Habit formation;  Habit reversal |
|  | “4. Time to stretch: If you are using a computer, set an alarm to go off every 20 minutes. When it rings, stand up and stretch, reaching your arms as high up as you can a few times. Hold each stretch for 10 seconds.” | Standing (balance)  Stretching (flexibility) | Prompts/cues;  Restructuring the physical environment;  Instruction on how to perform the behaviour;  Habit formation |
|  | “5. Tiptoe through the queue: When waiting in a queue … stand on your tip toes and then drop back down onto your heels with a gentle bang and bounce. Use support if you need to.” | Standing (balance)  Stretching (flexibility) | Instruction on how to perform the behaviour;  Prompts/cues;  Habit formation |
|  | “6. Watch your step: Set a target of walking at least 1500 steps each day. This is equivalent to walking at a normal pace for about 30 minutes. You could start by aiming for 500 steps (10 minutes of walking), and gradually build it up over time. […] Use a step counter to record your steps. […] Look for opportunities to increase your steps, e.g.  – take the lift to one floor below your destination and walk up the last staircase;  - walk around your home when on the phone;  - park further away from the supermarket entrance;  - get off the bus a stop or two early;  - find a slightly longer route to get home;  - when meeting friends, go for a walk together rather than sitting down.” | Walking (aerobic) | Goal setting (behaviour);  Action planning;  Graded tasks;  Self-monitoring behaviour;  Behaviour substitution |
|  | “7. Sit to stand with no hands: Each time you stand up, try doing it without using your hands. Make sure your feet are flat on the floor and your chair is sturdy. […] As you get up, try holding your position a few inches above the chair and count to ten. You could also try standing up and then sitting back down again, gradually doing more as it becomes easier.” | Standing (balance)  Weight-bearing (muscle-strengthening) | Instruction on how to perform behaviour;  Graded tasks;  Habit formation |
|  | “8. Improve your posture: […] Stand with your back to the wall with your heels two inches from it. With your chin tucked in, move the back of your head towards the wall.” | Posture (flexibility) | Instruction on how to perform behaviour |
|  | “9. Limber up: Do these physical activities in the same order each morning, at your own pace:  9a. Calf stretch  9b. Chest stretch  9c. Toe rises  9d. Walk as if on a tightrope across the floor  9e. March on the spot  9f. Walk your fingers up the wall  9g. Lift a tin of food in each hand.  Handy hint: Put notes on your fridge, cupboard or wall to remind yourself to do your chosen activities.” ** | Stretching (flexibility)  Stretching (flexibility)  Stretching (flexibility)  Balance  Marching (aerobic)  Stretching (flexibility)  Weight-bearing (muscle-strengthening) | Instruction on how to perform behaviour;  Demonstration of behaviour;  Graded tasks;  Prompts/cues;  Restructuring the physical environment |
|  | “10. Wall push-ups: do 10-push ups against a wall each morning. […] As your arms strengthen, increase the number of push-ups you do, resting for 1-2 minutes after every 10 push-ups.” | Weight-bearing (muscle-strengthening) | Instruction on how to perform behaviour;  Demonstration of behaviour;  Graded tasks |
| Habit-formation advice | Importance of:  - planning how, when and where to enact behaviour.  - increasing activity intensity gradually.  - repeating behaviour in stable settings  - continuing repetition after missed opportunities  - self-monitoring performance | N/A | Action planning;  Graded tasks;  Habit formation;  Self-monitoring behaviour |
| Misc advice | - Respond to urges to sit by reviewing whether “you have done enough to have earned that seat”, i.e. at least 10 consecutive minutes of activity.  - Monitor improvements in physical functioning arising from adherence to tips. | N/A | Framing/reframing;  Prompts/cues;  Goal setting (behaviour);  Self-monitoring outcome of behaviour |
| Supplementary tick-sheets | Daily self-monitoring record | N/A | Self-monitoring behaviour;  Self-monitoring outcome of behaviour |

* Tip descriptions are not comprehensive. Only text explicitly describing a behaviour change recommendation is included in this table; justifications or explanation of tips are not provided. ** Activities outlined in Tips 9a-9g and Tip 10 were outlined with extensive instructions and photographs, modelled by a female aged 66 years, to illustrate ergonomically correct procedures.
