# Supplementary material for: Acceptability of a theory-based sedentary behaviour reduction intervention for older adults (‘On Your Feet to Earn Your Seat’)
Source: BMC Public Health. 2015 Jul 2;15:606. doi: 10.1186/s12889-015-1921-0 (PMC4489366; doi:10.1186/s12889-015-1921-0)
Supplement: Additional file 2: Table S2. — Mean total per-week adherence to intervention tips, Weeks 2–8, Samples 1 and 2, completers only. [file 12889_2015_1921_MOESM2_ESM.docx]

**Additional file 2: Table S2.** Mean total per-week adherence to intervention tips, Weeks 2-8, Samples 1 and 2.

| *Tips* | *Week* | *Adherence* | | | | | |
| --- | --- | --- | --- | --- | --- | --- | --- |
|  |  | *Sample 1* | | | *Sample 2* | | |
|  |  | *N* | *Range* | *Mean adherence*  *(SD)* | *N* | *Range* | *Mean adherence*  *(SD)* |
| “1. Leave the house daily: Ensure that you go out at least once a day.” | Week (W) 2 | 11 | 0-100% | 59.74%  (35.47) | 22 | 0-100% | 82.47%  (26.43) |
|  | W3 | 11 | 0-100% | 50.65%  (42.07) | 22 | 0-100% | 85.06%  (26.63) |
|  | W4 | 11 | 0-100% | 59.74%  (42.77) | 22 | 14-100% | 85.71%  (25.33) |
|  | W5 | 11 | 0-100% | 54.55%  (41.81) | 22 | 14-100% | 83.12%  (23.59) |
|  | W6 | 11 | 0-100% | 49.35%  (45.34) | 21 | 0-100% | 80.95%  (28.33) |
|  | W7 | 11 | 0-100% | 45.45%  (45.54) | 21 | 0-100% | 75.51%  (32.31) |
|  | W8 | 10 | 0-100% | 40.00%  (40.85) | 21 | 0-100% | 71.43%  (37.25) |
| “2. Make ad breaks active: When you watch TV, stand up or walk around during breaks between programmes.” | W2 | 11 | 0-100% | 67.53%  (45.22) | 21 | 0-100% | 71.43%  (36.70) |
|  | W3 | 11 | 0-100% | 59.74%  (49.00) | 21 | 0-100% | 66.67%  (42.94) |
|  | W4 | 11 | 0-100% | 66.23%  (44.39) | 21 | 0-100% | 60.54%  (40.63) |
|  | W5 | 11 | 0-100% | 62.34%  (44.85) | 21 | 0-100% | 55.10%  (40.48) |
|  | W6 | 11 | 0-100% | 64.94%  44.89 | 20 | 0-100% | 60.71%  (46.32) |
|  | W7 | 11 | 0-100% | 59.74%  (45.99) | 20 | 0-100% | 62.86%  (41.30) |
|  | W8 | 11 | 0-100% | 45.45%  (45.54) | 20 | 0-100% | 50.71%  (45.76) |
| “3. Take a stand: Stand up when waiting for a bus or train.” | W2 | 11 | 0-100% | 37.66%  (49.60) | 22 | 0-100% | 66.23%  (38.06) |
|  | W3 | 11 | 0-100% | 44.16%  (45.83) | 22 | 0-100% | 67.53%  (43.68) |
|  | W4 | 11 | 0-100% | 42.86%  (47.38) | 22 | 0-100% | 61.04%  (43.46) |
|  | W5 | 11 | 0-100% | 41.56%  (43.54) | 22 | 0-100% | 58.44%  (43.62) |
|  | W6 | 11 | 0-100% | 40.26%  (44.18) | 21 | 0-100% | 61.22%  (43.36) |
|  | W7 | 11 | 0-100% | 37.66%  (41.54) | 21 | 0-100% | 63.95%  (43.48) |
|  | W8 | 11 | 0-100% | 27.27%  (39.62) | 21 | 0-100% | 59.18%  (41.47) |
| “4. Time to stretch: If you are using a computer, set an alarm to go off every 20 minutes. When it rings, stand up and stretch.” | W2 | 11 | 0-100% | 64.94%  (43.97) | 22 | 0-100% | 60.39%  (42.27) |
|  | W3 | 11 | 0-100% | 49.35%  (47.11) | 22 | 0-100% | 61.69%  (40.98) |
|  | W4 | 11 | 0-100% | 40.26%  (47.30) | 22 | 0-100% | 64.29%  (41.06) |
|  | W5 | 11 | 0-100% | 45.45%  (46.43) | 22 | 0-100% | 58.44%  (41.10) |
|  | W6 | 11 | 0-100% | 41.56%  (49.26) | 21 | 0-100% | 63.27%  (43.70) |
|  | W7 | 11 | 0-100% | 37.66%  (45.75) | 21 | 0-100% | 63.27%  (41.05) |
|  | W8 | 11 | 0-100% | 23.38%  (39.52) | 21 | 0-100% | 52.38%  (44.80) |
| “5. Tiptoe through the queue: When waiting in a queue … stand on your tip toes and then drop back down onto your heels gently.” | W2 | 11 | 0-100% | 35.06%  (42.55) | 22 | 0-100% | 41.56%  (39.16) |
|  | W3 | 11 | 0-100% | 19.48%  (35.15) | 22 | 0-100% | 42.21%  (41.23) |
|  | W4 | 11 | 0-86% | 14.29%  (31.94) | 22 | 0-100% | 35.71%  (42.23) |
|  | W5 | 11 | 0-100% | 12.99% | 22 | 0-100% | 35.06%  (38.62) |
|  | W6 | 11 | 0-43% | 7.79%  (17.34) | 21 | 0-100% | 31.97%  (38.04) |
|  | W7 | 11 | 0-43% | 6.49%  (14.80) | 21 | 0-100% | 38.78%  (40.19) |
|  | W8 | 11 | 0-57% | 7.79%  (18.48) | 21 | 0-100% | 40.14%  (41.56) |
| “6. Watch your step: Set a target of walking at least 1500 steps each day.” | W2 | 11 | 0-100% | 44.16%  (45.83) | 22 | 0-100% | 50.00%  (43.59) |
|  | W3 | 11 | 0-100% | 45.45%  (48.16) | 22 | 0-100% | 46.75%  (45.00) |
|  | W4 | 11 | 0-100% | 50.65%  (50.05) | 22 | 0-100% | 48.70%  (45.74) |
|  | W5 | 11 | 0-100% | 48.05%  (47.50) | 22 | 0-100% | 45.45%  (42.43) |
|  | W6 | 11 | 0-100% | 44.16%  (47.15) | 21 | 0-100% | 48.30%  (42.49) |
|  | W7 | 11 | 0-100% | 31.17%  (45.99) | 21 | 0-100% | 45.58%  (44.41) |
|  | W8 | 11 | 0-100% | 19.48%  (32.75) | 21 | 0-100% | 51.02%  (47.50) |
| “7. Sit to stand with no hands: Each time you stand up, try doing it without using your hands.” | W2 | 11 | 0-100% | 54.55%  (47.30) | 22 | 14-100% | 82.47%  (26.43) |
|  | W3 | 11 | 0-100% | 61.04%  (44.76) | 22 | 14-100% | 83.77%  (25.44) |
|  | W4 | 11 | 0-100% | 57.14%  (42.86) | 22 | 0-100% | 74.68%  (37.39) |
|  | W5 | 11 | 0-100% | 51.95%  (46.19) | 22 | 0-100% | 62.99%  (42.67) |
|  | W6 | 11 | 0-100% | 45.45%  (46.43) | 21 | 0-100% | 68.03%  (41.13) |
|  | W7 | 11 | 0-100% | 48.05%  (46.19) | 21 | 0-100% | 68.03%  (40.13) |
|  | W8 | 11 | 0-100% | 29.87%  (38.04) | 21 | 0-100% | 70.75%  (41.28) |
| “8. Improve your posture: Stand with your back to the wall with your heels two inches from it … and move the back of your head towards the wall.” | W2 | 11 | 0-100% | 38.96%  (46.55) | 22 | 0-100% | 62.34%  (39.32) |
|  | W3 | 11 | 0-100% | 42.86%  (49.49) | 22 | 0-100% | 61.69%  (41.91) |
|  | W4 | 11 | 0-100% | 41.56%  (48.43) | 22 | 0-100% | 64.94%  (43.58) |
|  | W5 | 11 | 0-100% | 37.66%  (40.54) | 22 | 0-100% | 49.35%  (41.99) |
|  | W6 | 11 | 0-100% | 40.26%  (49.00) | 21 | 0-100% | 50.34%  (42.04) |
|  | W7 | 11 | 0-100% | 35.06%  (45.34) | 21 | 0-100% | 55.78%  (47.58) |
|  | W8 | 11 | 0-100% | 27.27%  (44.01) | 21 | 0-100% | 48.30%  (45.73) |
| “9. Limber up: |  |  |  |  |  |  |  |
| 9a. Calf stretch | W2 | 11 | 0-100% | 54.55%  (52.22) | 22 | 0-100% | 66.88%  (44.17) |
|  | W3 | 11 | 0-100% | 53.25%  (51.15) | 22 | 0-100% | 64.94%  (43.81) |
|  | W4 | 11 | 0-100% | 61.04%  (48.69) | 22 | 0-100% | 59.74%  (45.31) |
|  | W5 | 11 | 0-100% | 48.05%  (50.42) | 22 | 0-100% | 54.55%  (45.95) |
|  | W6 | 11 | 0-100% | 50.65%  (50.05) | 21 | 0-100% | 49.66%  (42.77) |
|  | W7 | 11 | 0-100% | 57.14%  (49.90) | 21 | 0-100% | 57.14%  (45.18) |
|  | W8 | 11 | 0-100% | 41.56%  (47.58) | 21 | 0-100% | 54.42%  (47.73) |
| 9b. Chest stretch | W2 | 11 | 0-100% | 62.34%  (49.60) | 22 | 0-100% | 63.64%  (39.12) |
|  | W3 | 11 | 0-100% | 58.44%  (48.00) | 22 | 0-100% | 61.69%  (42.83) |
|  | W4 | 11 | 0-100% | 51.95%  (45.75) | 22 | 0-100% | 69.48%  (37.74) |
|  | W5 | 11 | 0-100% | 58.44%  (44.93) | 22 | 0-100% | 57.79%  (38.81) |
|  | W6 | 11 | 0-100% | 53.25%  (47.85) | 21 | 0-100% | 61.90%  (41.24) |
|  | W7 | 11 | 0-100% | 58.44%  (49.26) | 21 | 0-100% | 59.18%  (42.45) |
|  | W8 | 11 | 0-100% | 40.26%  (48.16) | 21 | 0-100% | 54.42%  (45.09) |
| 9c. Toe rises | W2 | 11 | 0-100% | 55.84%  (47.15) | 22 | 0-100% | 69.48%  (34.23) |
|  | W3 | 11 | 0-100% | 50.65%  (48.81) | 22 | 0-100% | 70.13%  (37.12) |
|  | W4 | 11 | 0-100% | 51.95%  (50.01) | 22 | 0-100% | 62.99%  (41.51) |
|  | W5 | 11 | 0-100% | 49.35%  (48.81) | 22 | 0-100% | 59.09%  (40.72) |
|  | W6 | 11 | 0-100% | 55.84%  (47.58) | 21 | 0-100% | 58.50%  (39.62) |
|  | W7 | 11 | 0-100% | 44.16%  (50.89) | 21 | 0-100% | 60.54%  (38.57) |
|  | W8 | 11 | 0-100% | 23.38%  (39.52) | 21 | 0-100% | 59.18%  (41.72) |
| 9d. Walk as if on a tightrope across the floor | W2 | 11 | 0-100% | 44.16%  (44.93) | 22 | 0-100% | 58.44%  (41.57) |
|  | W3 | 11 | 0-100% | 37.66%  (45.75) | 22 | 0-100% | 59.74%  (42.43) |
|  | W4 | 11 | 0-100% | 45.45%  (45.09) | 22 | 0-100% | 52.60%  (46.11) |
|  | W5 | 11 | 0-100% | 42.86%  (48.23) | 22 | 0-100% | 43.51%  (41.94) |
|  | W6 | 11 | 0-100% | 35.06%  (44.89) | 21 | 0-100% | 40.82%  (44.56) |
|  | W7 | 11 | 0-100% | 27.27%  (46.71) | 21 | 0-100% | 38.10%  (41.24) |
|  | W8 | 11 | 0-100% | 18.18%  (32.63) | 21 | 0-100% | 37.41%  (41.77) |
| 9e. March on the spot | W2 | 11 | 0-100% | 51.95%  (48.77) | 22 | 0-100% | 65.58%  (41.98) |
|  | W3 | 11 | 0-100% | 50.65%  (48.77) | 22 | 0-100% | 66.88%  (42.83) |
|  | W4 | 11 | 0-100% | 41.56%  (46.71) | 22 | 0-100% | 54.55%  (46.58) |
|  | W5 | 11 | 0-100% | 35.06%  (45.34) | 22 | 0-100% | 50.65%  (43.81) |
|  | W6 | 11 | 0-100% | 32.47%  (46.55) | 21 | 0-100% | 51.70%  (44.83) |
|  | W7 | 11 | 0-100% | 35.06%  (44.89) | 21 | 0-100% | 46.26%  (46.71) |
|  | W8 | 11 | 0-100% | 15.58%  (30.27) | 21 | 0-100% | 44.90%  (43.40) |
| 9f. Walk your fingers up the wall | W2 | 11 | 0-100% | 48.05%  (47.93) | 22 | 0-100% | 57.79%  (42.40) |
|  | W3 | 11 | 0-100% | 45.45%  (49.82) | 22 | 0-100% | 56.49%  (43.75) |
|  | W4 | 11 | 0-100% | 37.66%  (44.39) | 22 | 0-100% | 50.00%  (44.69) |
|  | W5 | 11 | 0-100% | 32.47%  (46.55) | 22 | 0-100% | 44.81%  (41.19) |
|  | W6 | 11 | 0-100% | 23.38%  (41.54) | 21 | 0-100% | 44.22%  (42.11) |
|  | W7 | 11 | 0-100% | 27.27%  (46.71) | 21 | 0-100% | 42.86%  (43.10) |
|  | W8 | 11 | 0-71% | 12.99%  (25.92) | 21 | 0-100% | 36.73%  (42.03) |
| 9g. Lift a tin of food in each hand.” | W2 | 11 | 0-100% | 44.16%  (46.27) | 22 | 0-100% | 46.75%  (46.07) |
|  | W3 | 11 | 0-100% | 48.05%  (44.85) | 22 | 0-100% | 43.51%  (41.23) |
|  | W4 | 11 | 0-100% | 44.16%  (47.15) | 22 | 0-100% | 48.05%  (46.35) |
|  | W5 | 11 | 0-100% | 48.05%  (44.85) | 22 | 0-100% | 38.96%  (43.91) |
|  | W6 | 11 | 0-100% | 50.65%  (50.05) | 21 | 0-100% | 38.10%  (41.49) |
|  | W7 | 11 | 0-100% | 53.25%  (51.15) | 21 | 0-100% | 34.01%  (41.52) |
|  | W8 | 11 | 0-100% | 18.18%  (31.36) | 21 | 0-100% | 31.29%  (39.54%) |
| “10. Wall push-ups: do 10-push ups against a wall each morning.” | W2 | 11 | 0-100% | 61.04%  (49.11) | 22 | 0-100% | 68.83%  (40.08) |
|  | W3 | 11 | 0-100% | 62.34%  (49.60) | 22 | 0-100% | 65.58%  (37.58) |
|  | W4 | 11 | 0-100% | 54.55%  (45.99) | 22 | 0-100% | 68.18%  (39.42) |
|  | W5 | 11 | 0-100% | 48.05%  (43.93) | 22 | 0-100% | 57.79%  (38.05) |
|  | W6 | 11 | 0-100% | 48.05%  (47.93) | 21 | 0-100% | 57.14%  (40.66) |
|  | W7 | 11 | 0-100% | 49.35%  (48.81) | 21 | 0-100% | 51.02%  (41.55) |
|  | W8 | 11 | 0-100% | 28.57%  (39.90) | 21 | 0-100% | 50.34%  (42.29) |

SD = Standard deviation, W = week number.
