# Supplementary material for: Acceptability of a theory-based sedentary behaviour reduction intervention for older adults (‘On Your Feet to Earn Your Seat’)
Source: BMC Public Health. 2015 Jul 2;15:606. doi: 10.1186/s12889-015-1921-0 (PMC4489366; doi:10.1186/s12889-015-1921-0)
Supplement: Additional file 3: Table S3. — Mean and median weekly adherence across all tips, Weeks 2–8, Samples 1 and 2, completers only. [file 12889_2015_1921_MOESM3_ESM.docx]

**Additional file 3: Table S3.** Mean and median weekly adherence across all tips, Weeks 2-8, Samples 1 and 2.

| *Week* | *Sample 1* | | | | *Sample 2* | | | |
| --- | --- | --- | --- | --- | --- | --- | --- | --- |
|  | *N* | *Observed range* | *Mean*  *(SD)* | *Median* | *N* | *Observed range* | *Mean*  *(SD)* | *Median* |
| Week 2 | 11 | 0-88% | 51.54%  (32.50) | 58.04% | 21 | 10-84% | 64.80%  (24.85) | 69.64% |
| Week 3 | 11 | 0-86% | 48.70%  (33.17) | 54.46% | 21 | 8-86% | 64.07%  (26.53) | 69.64% |
| Week 4 | 11 | 0-84% | 47.56%  (31.44) | 58.04% | 21 | 9-91% | 61.10%  (27.98) | 66.96% |
| Week 5 | 11 | 0-87% | 44.81%  (31.00) | 43.75% | 21 | 7-87% | 54.29%  (26.95) | 54.46% |
| Week 6 | 11 | 0-82% | 42.69%  (33.16) | 43.75% | 20 | 0-100% | 55.22%  (29.68) | 52.68% |
| Week 7 | 11 | 0-75% | 40.83%  (31.76) | 43.75% | 20 | 0-94% | 54.64%  (28.43) | 55.80% |
| Week 8 | 10 | 0-75% | 28.57%  (28.66) | 20.54% | 20 | 0-100% | 51.83%  (31.47) | 45.98% |
