# Supplementary material for: Acceptability of a theory-based sedentary behaviour reduction intervention for older adults (‘On Your Feet to Earn Your Seat’)
Source: BMC Public Health. 2015 Jul 2;15:606. doi: 10.1186/s12889-015-1921-0 (PMC4489366; doi:10.1186/s12889-015-1921-0)
Supplement: Additional file 4: Table S4. — Changes in physical activity, sedentary behaviour and habit from T1, Samples 1 and 2, completers only. [file 12889_2015_1921_MOESM4_ESM.docx]

**Additional file 4: Table S4**. Changes in physical activity, sedentary behaviour and habit from T1, Samples 1 and 2.

|  | *Change in behaviour/habit from T1* | | | | | |
| --- | --- | --- | --- | --- | --- | --- |
|  | *Sample 1* | | | *Sample 2* | | |
|  | *Direction of change*  *(observed range of change scores)** | *T2*  *N (%)* | *T3*  *N (%)* | *Direction of change*  *(observed range of change scores) ** | *T2*  *N (%)* | *T3*  *N (%)* |
| ***Sedentary behaviour*** |  |  |  |  |  |  |
| Sitting time (IPAQ), mins/week |  | *N = 9* | *N = 10* |  | *N = 23* | *N = 23* |
|  | Increase  *(≥ 210 ≤ 2730mins)* | 2  (22%) | 4  (40%) | Increase  *(≥ 210 ≤ 1050mins)* | 5  (22%) | 5  (22%) |
|  | No change  *(0 mins)* | 2  (22%) | 2  (10%) | No change  *(0 mins)* | 2  (9%) | 0  (0%) |
|  | Decrease  *(≤ -150 ≥ -1320mins)* | 5  (56%) | 4  (40%) | Decrease  *(≤ -240 ≥ -3300mins)* | 16  (70%) | 18  (78%) |
| Sitting time (MOST), mins/week |  | *N = 10* | *N = 10* |  | *N = 22* | *N = 23* |
|  | Increase  *(≥ 240 ≤ 4920mins)* | 4  (40%) | 6  (60%) | Increase  *(≥ 60 ≤ 2400mins)* | 9  (41%) | 10  (43%) |
|  | No change  *(0 mins)* | 0  (0%) | 0  (0%) | No change  *(0 mins)* | 0  (0%) | 0  (0%) |
|  | Decrease  *(≤ -120 ≥ -4313mins)* | 6  (60%) | 4  (40%) | Decrease  *(≤ -20 ≥ -6300mins)* | 13  (59%) | 13  (57%) |
| Sitting habit  (1–7, 7= strongest habit) |  | *N = 11* | *N = 11* |  | *N = 22* | *N = 22* |
|  | Increase  *(≥ 0.25 ≤ 1.00)* | 2  (18%) | 3  (27%) | Increase  *(≥ 0.25 ≤ 2.25)* | 8  (36%) | 5  (23%) |
|  | No change  *(0)* | 6  (55%) | 4  (36%) | No change  *(0)* | 5  (23%) | 7  (32%) |
|  | Decrease  *(≤ -0.50 ≥ -1.00)* | 3  (27%) | 4  (36%) | Decrease  *(≤ -0.25 ≥ -3.25)* | 9  (41%) | 10  (46%) |
| ***Physical activity*** |  |  |  |  |  |  |
| Walking, mins/week |  | *N = 12* | *N = 12* |  | *N = 23* | *N = 23* |
|  | Increase  *(≥ 40 ≤ 3060mins)* | 9  (75%) | 6  (50%) | Increase  *(≥ 10 ≤ 1680mins)* | 15  (65%) | 17  (74%) |
|  | No change  *(0 mins)* | 1  (8%) | 2  (17%) | No change  *(0 mins)* | 2  (9%) | 1  (4%) |
|  | Decrease  *(≤ -40 ≥ -780mins)* | 2  (17%) | 4  (33%) | Decrease  *(≤ -30 ≥ -1020mins)* | 6  (26%) | 4  (17%) |
| Moderate PA, mins/week |  | *N = 12* | *N = 12* |  | *N = 23* | *N = 23* |
|  | Increase  *(≥ 10 ≤ 150mins)* | 3  (25%) | 2  (17%) | Increase  *(≥ 20 ≤ 720mins)* | 8  (35%) | 13  (56%) |
|  | No change  *(0 mins)* | 7  (58%) | 8  (67%) | No change  *(0 mins)* | 6  (26%) | 5  (22%) |
|  | Decrease  *(≤ -60 ≥ -240mins)* | 2  (17%) | 2  (17%) | Decrease  *(≤ -30 ≥ -1680mins)* | 9  (39%) | 5  (22%) |
| Vigorous PA, mins/week |  | *N = 12* | *N = 12* |  | *N = 23* | *N = 23* |
|  | Increase  *(≥ 20 ≤ 660mins)* | 0  (0%) | 2  (17%) | Increase  *(≥ 45 ≤ 900mins)* | 7  (30%) | 13  (56%) |
|  | No change  *(0 mins)* | 11  (92%) | 9  (75%) | No change  *(0 mins)* | 12  (52%) | 7  (30%) |
|  | Decrease  *(= -60mins)* | 1  (8%) | 1  (8%) | Decrease  *(≤ -3 ≥ -180mins)* | 4  (17%) | 3  (13%) |
| PA habit  (1–7, 7= strongest habit) |  | *N = 11* | *N = 11* |  | *N = 23* | *N = 23* |
|  | Increase  *(≥ 0.25 ≤ 2.75)* | 3  (27%) | 6  (55%) | Increase  *(≥ 0.25 ≤ 3.00)* | 13  (56%) | 12  (52%) |
|  | No change  *(0)* | 2  (18%) | 1  (9%) | No change  *(0)* | 5  (22%) | 3  (13%) |
|  | Decrease  *(≤ -0.50 ≥ -2.50)* | 6  (55%) | 4  (36%) | Decrease  *(≤ -0.25 ≥ -1.50)* | 6  (26%) | 8  (17%) |

* All difference scores other than zero were treated as increases or decreases. Ranges represent *actual* minimum and maximum positive and negative changes in study variables.
